# Protocol for quantifying muscle fiber size, number, and central nucleation of mouse skeletal muscle cross-sections using Myotally software

**DOI:** 10.1016/j.xpro.2024.103555

**Published:** 2025-01-11

**Authors:** Pieter Both, Soochi Kim, Jengmin Kang, Marina Arjona, Daniel I. Benjamin, Christopher W. Nutter, Armon Goshayeshi, Thomas A. Rando

**Affiliations:** 1 Department of Neurology and Neurological Sciences, Stanford University School of Medicine, Stanford, CA 94305, USA; 2 Stem Cell Biology and Regenerative Medicine Graduate Program, Stanford University School of Medicine, Stanford, CA 94305, USA; 3 Department of Biotechnology and Bioinformatics, Korea University, Sejong 30019, Republic of Korea; 4 Neurology Service, Veterans Affairs Palo Alto Health Care System, Palo Alto, CA 94304, USA

**Keywords:** Cell Biology, Microscopy, Computer sciences

## Abstract

Here, we present a protocol for using Myotally, a user-friendly software for fast, automated quantification of muscle fiber size, number, and central nucleation from immunofluorescent stains of mouse skeletal muscle cross-sections. We describe steps for installing the software, preparing compatible images, finding the file path, and selecting key parameters like image quality and size limits. We also detail optional features, such as measuring mean fluorescence. By automating these traditionally labor-intensive processes, Myotally improves research efficiency and data consistency.

## Highlights

• Protocol quantifying muscle fiber size, number, and central nucleation
• Steps for software installation and image preparation
• Instructions for selecting file path and setting key parameters
• Guidance on measuring mean fluorescence intensity

## Abstract (editor highlights)

Publisher’s note: Undertaking any experimental protocol requires adherence to local institutional guidelines for laboratory safety and ethics.

## Graphical abstract

**Figure:**
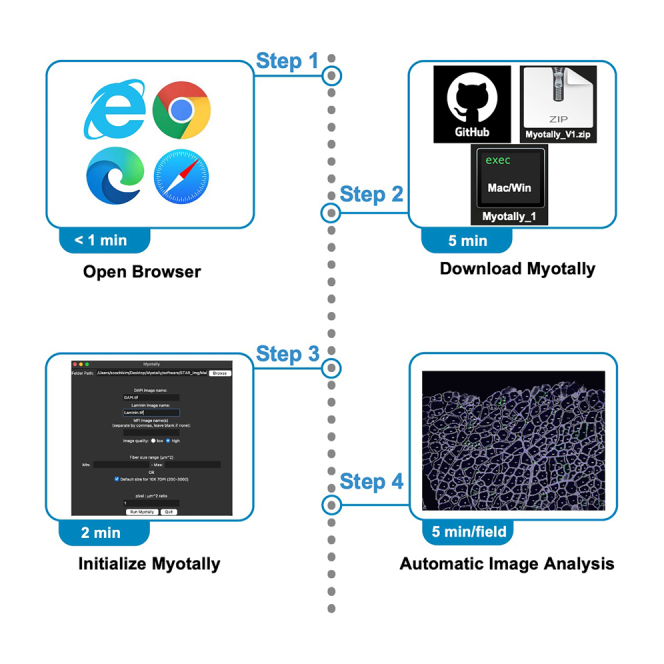

## Before you begin

Muscle is a highly regenerative tissue with the capacity to fully repair itself even after repeated bouts of injury, an ability conferred by tissue resident muscle stem cells (MuSCs) and the supporting niche.^1^^,^^2^ Though quiescent under homeostatic conditions, MuSCs activate in response to signaling cues from injured muscle, proliferate, differentiate, and fuse to form nascent myotubes.^3^^,^^4^ The phases of muscle regeneration can be detected by the expression of myogenic transcription factors, such as MyoD and Myogenin in activated MuSCs and differentiating myoblasts, respectively.^5^^,^^6^ Nascent myotubes begin to form within days of the onset of muscle regeneration and are characterized by centrally located nuclei and the expression of embryonic myosin heavy chain (eMyHC).^7^^,^^8^ Fluorescence intensity of individual myofibers based on the expression of eMyHC^9^ and the differential expression of MyHC isoforms^10^ are often used to assess the newly forming and distribution of myofiber types, respectively. Newly formed myotubes progressively undergo hypertrophy as they become mature myofibers, and their size, frequently measured as cross-sectional area (CSA) or minimal Feret diameter (MFD), at a given time after injury is used as a histological readout for the extent of muscle repair.^11^ Muscle regeneration can also be assessed using behavioral readouts such as gait analysis or treadmill performance, though these may be confounded by variables unrelated to muscle function. *Ex vivo* contractile force following electrical stimulation is a low throughput but more direct measurement of muscle function.

Myotally is a fully automated, Python-based software program that quantifies muscle fiber CSA, number, and central nucleation. Proof of concept data collected using ectopic RFP expression suggests that Myotally can also accurately quantify myofiber mean fluorescent intensity (MFI), though the efficacy of Myotally for detecting MFI of endogenous muscle proteins remains to be determined. This protocol outlines a stepwise quantification of myofiber size and number in both regenerating and uninjured muscle as well as an optional feature for quantifying fiber MFI. The optional MFI assessment is demonstrated using a Pax7^CreER/+;tdTomato^ mouse line, where MuSCs express a red fluorescent protein (RFP) lineage tracer and newly formed myofibers derived from these MuSCs also express RFPs.

### Institutional permissions

Animal experiments of muscle injury in mice using 1.2% BaCl_2_ (Sigma) are as previously described,^12^ approved by the Institutional Animal Care and Use Committee approved protocols at the Veterinary Medical Unit of the Veterans Affairs Palo Alto Health Care System (Protocol RAN1966).

### Tissue preparation

Injured and uninjured tibialis anterior (TA) muscles from wild-type mice were frozen immediately after dissection. TA muscles with RFP-expressing myofibers were fixed in 0.5% PFA and dehydrated in 20% sucrose prior to cryopreservation. Muscles were frozen in OCT (Fisher) by submersion in liquid nitrogen-cooled isopentane (Sigma) for 30 s and stored at −80°C. Cryopreserved TA muscles were sectioned at the mid-belly of the muscle at a thickness of 10 μm. Sections were fixed in 2% PFA at room temperature for 10 min and blocked overnight at 4°C in 2% BSA/0.3% PBS-Tween (Sigma). Blocking buffer was then removed and a primary antibody against Laminin (Abcam 11576) was added at a dilution of 1/1000 in blocking buffer at room temperature for 2 h. The primary antibody was then washed off with PBS and replaced by the secondary antibody (Thermo A32744) at a dilution of 1/500 and DAPI (Thermo D1306) at a dilution of 1/2000 in blocking buffer for 45 min. Slides were then washed with PBS, aspirated, and mounted with Fluorosave (Millipore) for imaging. Immunofluorescence images were acquired using an Observer Z1 fluorescent microscope (Carl Zeiss) and exported as .tif files with all channels separate before loading into the Myotally desktop application. Myotally is compatible with both MacOS (Monterey 12.4 or newer) and Windows (Win 10).

***Note:*** Images of muscle cross-sections used for input files should be stained for proteins that clearly delineate myofiber borders, such as Laminin or Dystrophin. While Myotally is compatible with various image qualities and common image formats, including .jpg., and .png., we strongly discourage the use of these formats for analysis. These lossy formats do not preserve critical metadata, including the effective pixel size (conversion factor or physical pixel size of the camera divided by total magnification). Without this information, quantification outputs may be inaccurate due to improper pixel-to-micrometer scaling.

Most color cameras have pixel sizes ranging from 3–5 μm, while fluorescence cameras often have 6.5 μm pixels. Depending on magnification (e.g., 10x or 20x), this results in effective pixel sizes ranging from 0.3 to 0.15 μm/pixel for color cameras or 0.65 to 0.325 μm/pixel for monochromatic cameras. We recommend using .tif, which is a lossless format that preserves pixel intensity values and ensure correct scaling. Always confirm your μm/pixel ratio before analysis. For optimal results, images with clear myofiber delineation in a lossless format will facilitate robust quantification. Therefore, this protocol assumes the user has performed a staining of myofiber borders of interest and done imaging using wide-field or confocal microscopy.

## Key resources table

**Table:** 

REAGENT or RESOURCE	SOURCE	IDENTIFIER
**Antibodies**		

Rat anti-Laminin 2 alpha (dilution 1:1,000)	Abcam	Cat# 11576; AB_298180
Donkey anti-rat Alexa Fluor 594 (dilution 1:500)	Thermo Fisher Scientific	Cat# A21209; AB_2535795

**Biological samples**		

TA muscle sections - uninjured (Benjamin et al.^12^) C57BL6 (12–16 week old, male)	JAX	000664
TA muscle sections – regenerating (7 DPI) (Benjamin et al.^12^) C57BL6 (12–16 week old, male)	JAX	000664
TA muscle sections – RFP labeled (Benjamin et al.^12^) Pax7CreER/+; ROSA26RFP (12–16 week old, female)	Dr. Charles Keller at Oregon Health & Science University, JAX, bred in-house	007914

**Chemicals, peptides, and recombinant proteins**		

Tamoxifen	Sigma-Aldrich	Cat# T5648
Corn oil	Sigma-Aldrich	Cat# C8767
BaCl_2_	Sigma-Aldrich	Cat# B0750
10X PBS	Thermo Fisher Scientific	Cat# mt-46-013-cm
16% Paraformaldehyde	Electron Microscopy Sciences	Cat# 15710
Sucrose	Fisher Scientific	Cat# bp220-1
Tissue-Tek OCT compound	Fisher	Cat# 14-373-65
DAPI (dilution 1:2,000)	Thermo Fisher Scientific	Cat# D1306
2-Methylbutane	Sigma-Aldrich	Cat# M32631
BSA	NEB	Cat# B9000S
Tween 20	Fisher BioReagents	Cat# BP337
FluorSave Reagent	Fisher	Cat# 345789

**Experimental models: Organisms/strains**		

Mouse: *Pax7*^*CreER*^	Nishijo et al.^13^	NA
Mouse: *ROSA26tdTomato*	The Jackson Laboratory	Cat# 007914; IMSR_JAX:007914

**Software and algorithms**		

Prism 10	GraphPad	https://www.graphpad.com/updates
Fiji	ImageJ	https://imagej.net/software/fiji/downloads
BioRender	BioRender	https://www.biorender.com/

## Materials and equipment

The Myotally desktop application can be used successfully with MacOS (Monterey 12.4 or newer) and Windows (Win 10). Software versions used to run the unpackaged Python scripts are Python 3.9.10, SciPy 1.8.0, NumPy 1.22.2, OpenCV 4.5.5, and Python Imaging Library (PIL) 9.0.1.

## Step-by-step method details

### Installation of Myotally desktop application

**Timing: 5 min**

In this step, install the Myotally desktop application and prepare sample image folders for automatic image analysis.

1. Download Myotally.
  a. Navigate to the following URLs:

  https://github.com/pieterboth/Myotally/releases/tag/Myotally_V1.0.1.

  https://zenodo.org/records/14197515.

**Figure 1:**
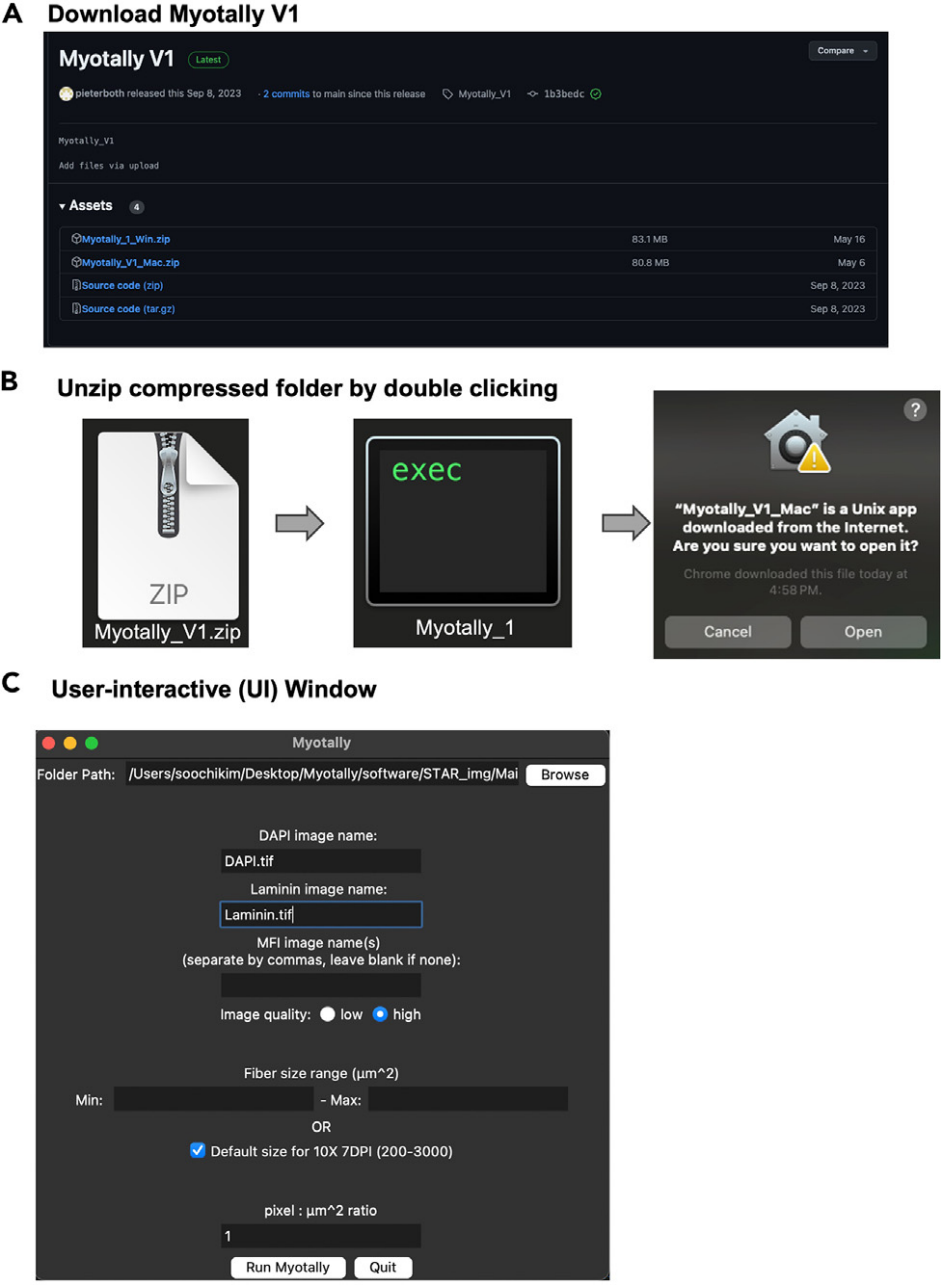
Instructions for using Myotally (A) Screenshot of Myotally GitHub page, to download Myotally V1. (B) Screenshots of the downloaded Myotally V1 for Mac and after unzip, and warning window for initialization. (C) Graphical user interface for entering input information to run Myotally.
3. Unzip the file (Figure 1B).
  a. Double-click the compressed folder to unzip it.

***Note:*** Myotally can be initialized by **right-clicking** on the application and selecting **‘Open’** (Double clicking on the icon may fail to open the application the first time but will work for subsequent attempts after you have opened Myotally once by right-clicking). You may then be warned that the identity of the developer cannot be confirmed; select **‘Open’**. A command prompt will immediately open, but no action is required here; this can be used to monitor the progress of image analysis.

2. Select your platform (Figure 1A).
  a. Download the appropriate file for your operating system:
    i. Myotally_V1_Mac.zip.
    ii. Myotally_V1_Win.zip.

For a step-by-step guide, you can also watch our video tutorial available at the following link: Myotally Video Tutorial.

4. Launch Myotally.

***Note:*** A user-interactive (UI) window will appear, prompting you to provide the location of the folder containing all the images to be analyzed.

### Preparing Myotally-compatible input images

**Timing: 5 min**

5. Convert ND2 or CZI files to stacked tiff images.
  a. Use the macro from this repository to convert your files:

  https://github.com/kalasNTY/ND2TIFF.

6. Open stacked tiff images in FIJI/ImageJ.
  a. In FIJI/ImageJ, go to File > Open and load your stacked tiff image.
7. Split channels (unmerging colors).
  a. Navigate to Image > Color > Split Channels to create individual images for each channel.
8. Saving individual channel images.
  a. Save each channel image in .tiff format (File > Save As >Tiff) and give them appropriate names (e.g., Laminin.tif, DAPI. tif), saving them in the chosen folder for analysis.

***Note:*** This macro works for both .nd2 and .czi formats.

### Myotally setup instructions

**Timing: 1 min**

9. Specify folder path using browse function.
  a. Use the Browse button in the Myotally interface to navigate and select the folder containing subfolders with images to analyze (Figure 1C).

**Figure 2:**
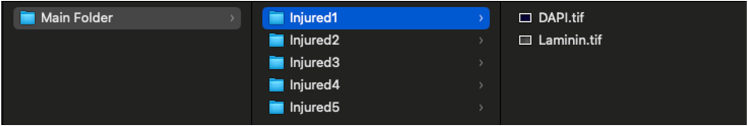
Example of how image folders and input images should be organized for single and batch processing

***Note:*** Images and folders can be named as desired, with the stipulation that channel names (e.g. DAPI.tif, Laminin.tif, RFP.tif) are consistent across all images (Figure 2). Each input image should be in its own folder, with corresponding channels in that folder, unmerged. All such image folders should be saved within a single main folder, for which the user will provide the pathname. Myotally also includes an optional feature to quantify MFI of fibers, which requires immunostaining of a protein of interest. In this protocol, we used RFP signals derived from Pax7^CreER/+;tdTomato^ mouse line, as a proof-of-principle.

### Choosing image quality

**Timing: 1 min**

10. Determine image quality.
  a. Classify image quality as either ‘low’ or ‘high’.

**Table 1:** Parameters for tissue imaging in Myotally

Parameter	Recommended range	Notes
Magnification	10x to 20x	Ensure clear visibility of myofiber outlines
Numerical Aperture (NA)	≥ 0.7	Higher NA improves resolution but reduces depth of field
Digital Resolution	1:1 μm/pixel	Sufficient for accurate segmentation
Focus	Good focus with low offset	Myofibers should be distinct and sharply focused
Depth of Field	Dependent on NA	Higher NA reduces depth of field but improves overall resolution

***Note:*** To determine image quality, consider the clarity and sharpness of the image. High-quality images will have clear, distinct boundaries between fibers and minimal background noise, and should be well-focused with a good depth of field. Low-quality images, on the other hand, will have blurred edges, poor contrast, and significant background interference. If the features of the fibers are difficult to distinguish or if there is a lot of noise, classify the image quality as ‘low’. Refer to the recommended parameters for tissue imaging in the Myotally (Table 1) below for further guidance.

### Choosing upper and lower size limits

**Timing: 1 min**

11. Set size limits.
  a. Set upper and lower size limits, in units of square microns (μm^2^), for fiber detection or select default size parameters. For images with a 1:1 pixel:μm^2^ ratio, accurate results are typically obtained using fiber size ranges of 200–4800 μm^2^ for uninjured TA muscles and 200–3000 μm^2^ for regenerating muscle at seven days post-injury (7 DPI).

***Note:*** The default size parameters are optimized for 7 DPI regenerating muscles, a common time point in muscle regeneration research. If images are taken at different magnifications, these size ranges should be adjusted empirically. Users should verify the pixel:μm^2^ ratio used by imaging software to ensure accurate image quantification. Fiber size ranges (μm^2^) can be determined empirically using programs like ImageJ to measure a few minimum and maximum fibers before setting the limits.

### Run Myotally

**Timing: 5 min per image/unspecified for batch processing**

12. Run Automatic Fiber Counts.
  a. Click ‘Run Myotally’ to begin the analysis.

***Note:*** Processing takes about 5 min per 10X field, and progress can be monitored in the command prompt.

## Expected outcomes

This protocol will enable users to obtain comprehensive quantification of muscle fiber size, number, and central nucleation from immunofluorescent labeled mouse skeletal muscle cross-sections with optional feature to measure MFI. The protocol provides a step-by-step visual guide from installation to application of Myotally.

**Figure 3:**
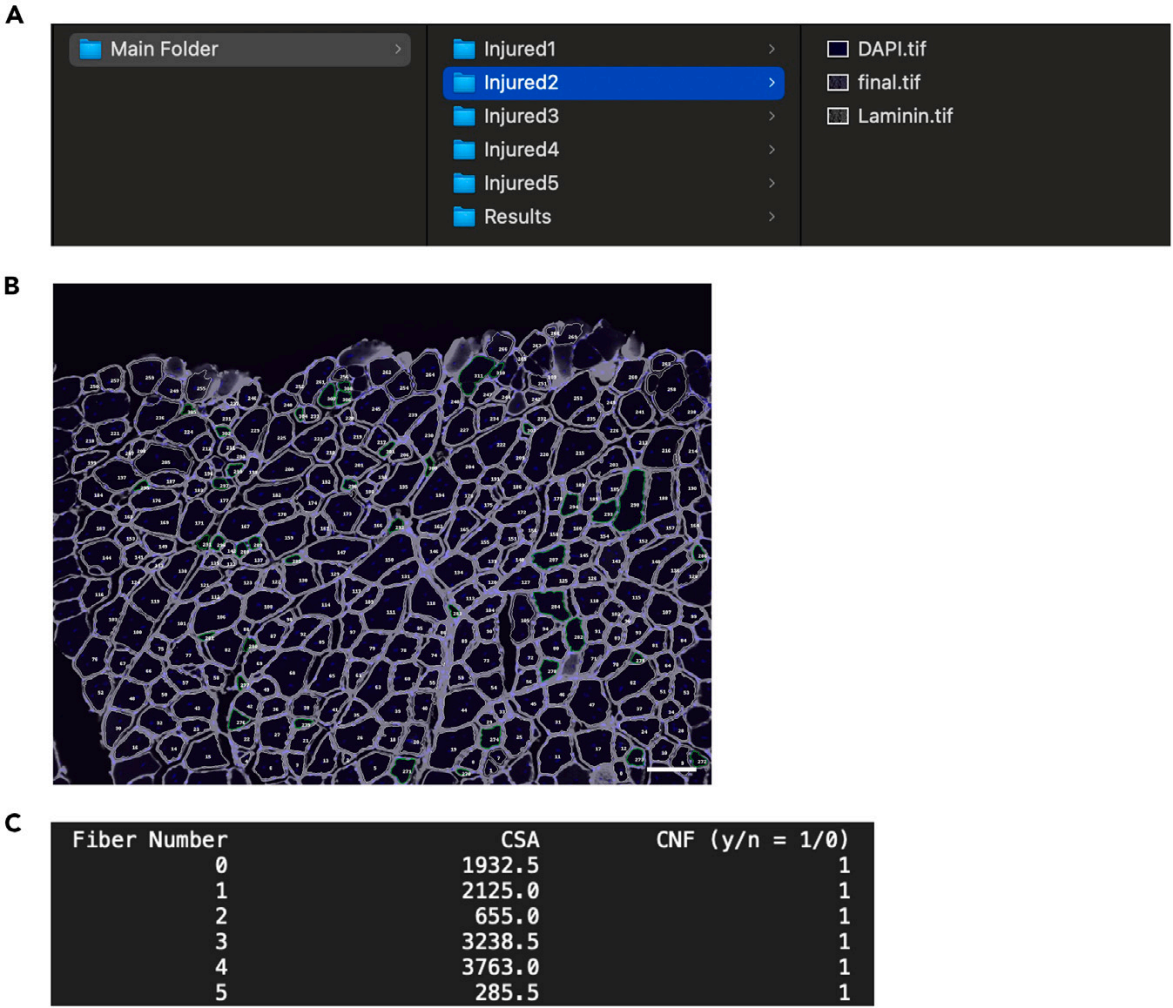
Screenshots of the Myotally outputs (A) Example of where an output ‘final.tif’ image file will be saved. (B) Representative image of ‘final.tif’ in which all measured fibers are numbered. (C) Sample.txt output file from automated image analysis that indexes myofiber number with CSA, and CNF.

Results are saved in text and image files, as each input image finishes processing. An output ‘final.tif’ image file, in which all measured fibers are numbered, will appear in each input image folder (Figures 3A and 3B). Quantitative results will appear in a ‘Results’ folder within the main folder and will include three text files per input image. Two will be CSV files that contain a comma separated list of CSAs and optionally, MFIs of all fibers measured in the corresponding image. CSA values are reported in units of μm^2^, and this number will be accurate only if users have specified the correct pixel to μm^2^ ratio used by their imaging software. The third text file will contain a numbered list of all fibers in the relevant input image together with the CSA and central nucleation status of each fiber, and optional MFIs (Figure 3C).

Users can cross reference this with the numbered ‘final.tif’ image to search for CSA, central nucleation, and MFI of any fiber from a given image.

## Quantification and statistical analysis

Myotally could be used in multiple experimental settings. In this protocol, we will demonstrate the automatic quantification of muscle fiber size, number, and central nucleation using Myotally. Additionally, we will cover an optional feature for measuring MFI, using a Pax7CreER-driven MuSC lineage tracer model.

**Figure 4:**
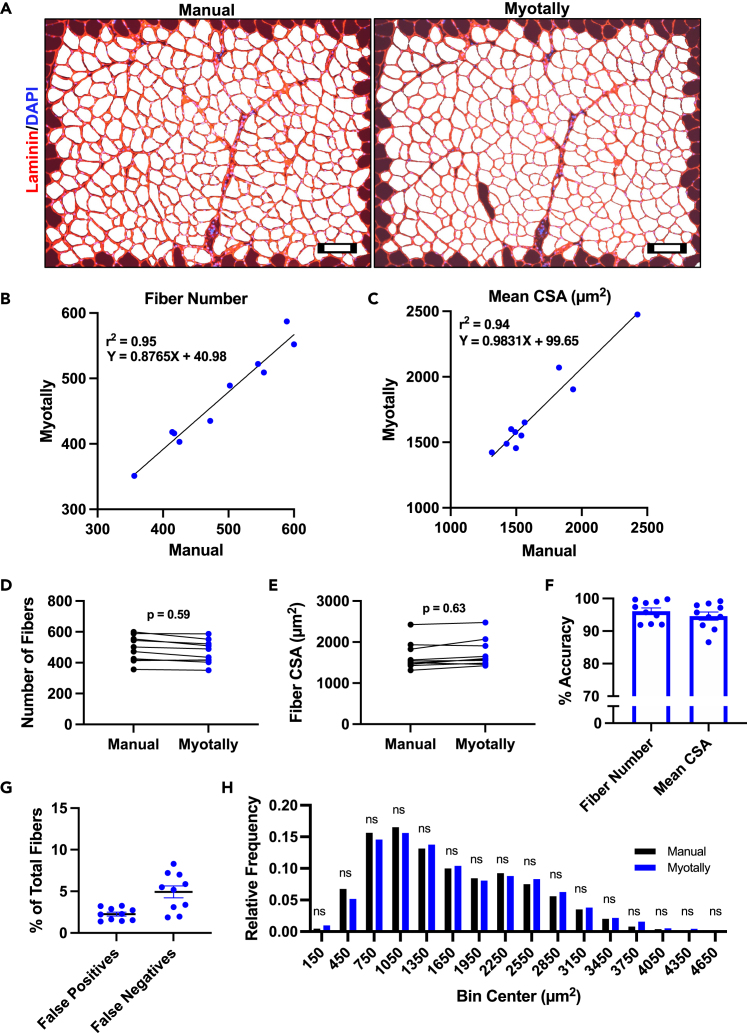
Manual validation of uninjured myofiber number and CSA measurements using Myotally (A) Representative images of uninjured TA cryosections that were stained for Laminin then analyzed for myofiber CSA manually using ImageJ (left) or automatically using Myotally (right). Scale bar, 100 μm. (B and C) Linear regression analysis between manually and automatically collected measurements of (B) myofiber number and (C) mean CSA from uninjured muscle. (D and E) Student’s t-test comparisons between manually and automatically collected measurements of (D) myofiber number and (E) mean CSA from uninjured muscle. (F) Percent accuracy of automatic measurements compared to manual measurements of myofiber number and mean CSA, calculated as $\%\;Accuracy=\frac{1-\left|Manual-Myotally\right|}{Manual}*100$. (G) Percent false positive and false negative myofiber counts by Myotally. (H) Frequency histogram of manually and automatically collected measurements of uninjured myofiber size, distributed into 300 μm^2^ bins. (*n* = 10 image fields); error bars represent SEM; ns = not significant; *p* values determined using Student’s t-tests.

To validate Myotally, we compared its measurements of myofiber number and cross-sectional area (CSA) against manual measurements from TA muscles of wild-type mice (Figure 4A). Linear regression analysis showed a high degree of statistical correlation between the methods, with no significant differences in fiber number and mean CSA (Figures 4B–4E). Myotally’s automatic measurements were 96.1% and 94.6% accurate for fiber number and size, respectively, compared to manual measurements (Figure 4F). Manual counting proved highly reproducible and low degree of operational error, with only 1.1% disagreement between different counts (Figure S1A). Myotally identified 2.3% false positive and 4.9% false negative fiber counts per image (Figure 4G). No significant differences in fiber size distributions were observed between manual and automatic measurements, confirming Myotally’s accuracy in uninjured tissue (Figure 4H).

**Figure 5:**
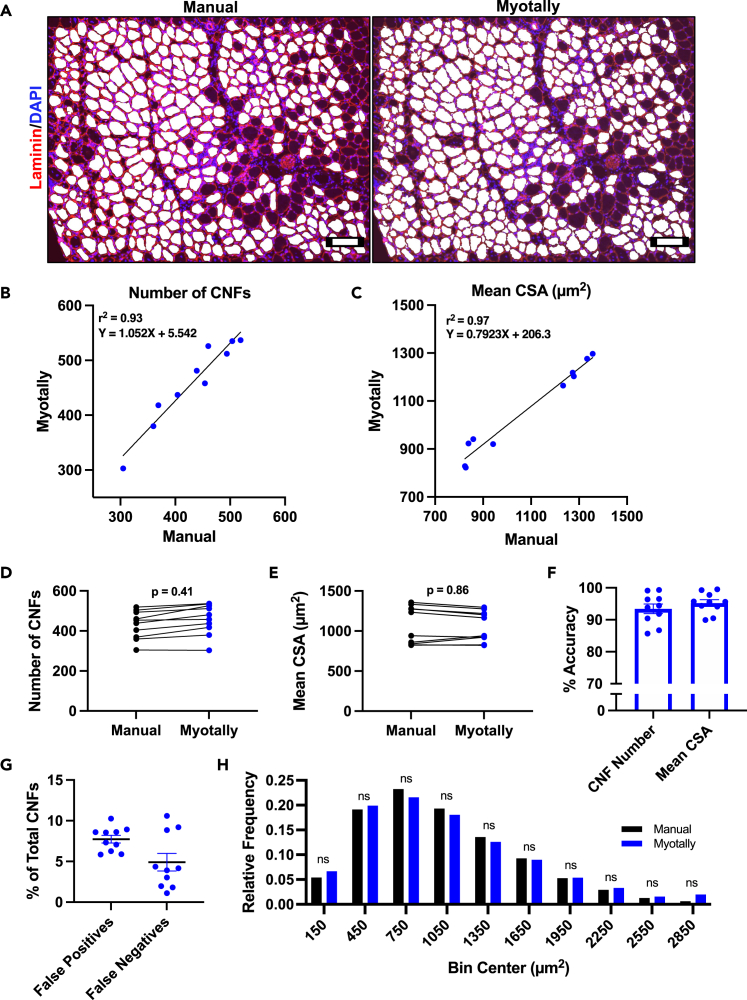
Manual validation of centrally nucleated myofiber number and CSA measurements using Myotally (A) Representative images of injured TA cryosections that were harvested seven days post injury (7 DPI), stained for Laminin, then analyzed for myofiber CSA manually using ImageJ (left) or automatically using Myotally (right). Scale bar, 100 μm. (B and C) Linear regression analysis between manually and automatically collected measurements of (B) centrally nucleated myofiber number and (C) mean CSA from 7 DPI muscle. (D and E) Student’s t-test between manually and automatically collected measurements of (D) centrally nucleated myofiber number and (E) mean CSA from 7 DPI muscle. (F) Percent accuracy of automatic measurements compared to manual measurements of centrally nucleated myofiber number and mean CSA, calculated as $\%\;Accuracy=\frac{1-\left|Manual-Myotally\right|}{Manual}*100$). (G) Percent false positive and false negative CNF counts by Myotally. (H) Frequency histogram of manually and automatically collected measurements of centrally nucleated myofiber size, distributed into 300 μm^2^ bins. (*n* = 10 image fields); error bars represent SEM; ns = not significant; *p* values determined using Student’s ttests.

For regenerating muscle, Myotally distinguished myofibers with central nuclei, a key feature of regenerating muscle fibers. We validated this by comparing manual and automatic measurements of central nucleated fibers (CNFs) from regenerating TA muscles (Figure 5A). Strong correlation was found between methods, with no significant differences in CNF number or mean CSA (Figures 5B–5E). Myotally’s measurements were 93.4% and 95.2% accurate for CNF number and mean CSA, respectively (Figure 5F). Manual CNF counting showed 6.6% disagreement between individuals (Figure S1B). Myotally had 7.7% false positive and 4.9% false negative CNF counts per image (Figure 5G). CNF size distributions were similar between methods (Figure 5H), validating Myotally for accurate and precise quantification of regenerating myofiber CSA with robust detection of central nucleation.

**Figure 6:**
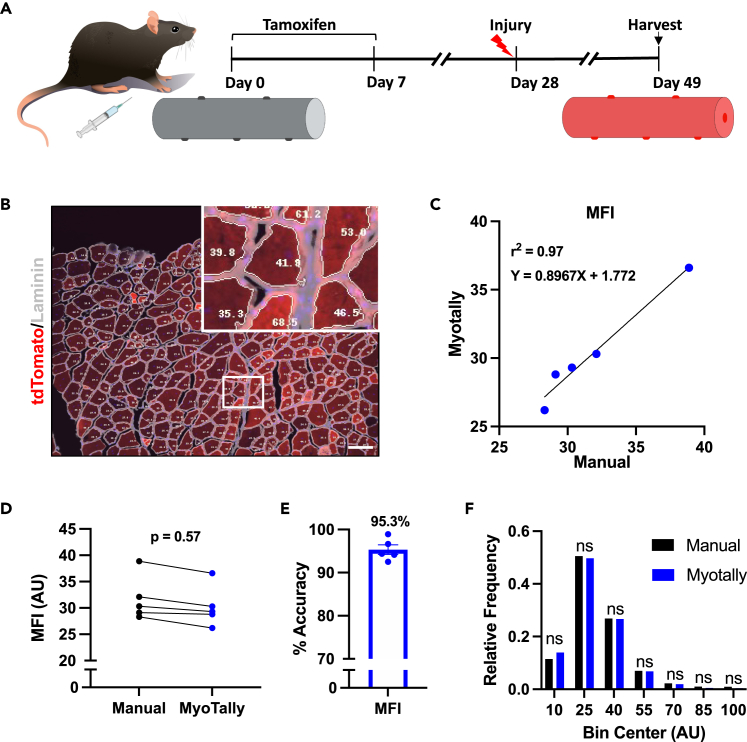
Manual validation of myofiber MFI measurements using Myotally (A) Diagram of tamoxifen treatment and injury timeline for generating RFP-labeled myofibers using a Pax7CreER-driven MuSC lineage tracer. (B) Representative image of RFP-labeled myofibers that were analyzed for MFI manually or automatically using Myotally. In the enlargement, fibers measured using Myotally are outlined in white with MFIs inscribed in corresponding fibers. Scale bar, 100 μm. (C) Linear regression analysis between manually and automatically collected measurements of MFI from RFP-labeled myofibers. (D) Student’s t-test comparison between manually and automatically collected measurements of MFI from RFP-labeled myofibers. (E) Percent accuracy of automatic measurements compared to manual measurements of MFI from RFP-labeled myofibers, calculated as $\%\;Accuracy=\frac{1-\left|Manual-Myotally\right|}{Manual}*100$). (F) Frequency histogram of manually and automatically collected measurements of MFI from RFP-labeled myofibers, distributed into 15 AU bins. (*n* = 5 image fields); error bars represent SEM; ns = not significant; *p* values determined using Student’s t-tests.

Additionally, Myotally has an optional feature to accurately quantify myofiber MFI, which can be used to reflect protein expression levels. As a proof of concept, we validated this feature using a Pax7^CreER/+;tdTomato^ mouse line, where MuSCs and their progeny expressed an RFP lineage tracer after tamoxifen treatment. Consequently, newly formed regenerating myofibers also express RFP, as demonstrated in Figure 6A. Myotally’s automatic MFI measurements were highly correlated with manual measurements (r^2^ = 0.97), with 95.3% accuracy and no significant differences observed (Figures 6B–6E). No significant differences in MFI distributions were found between methods, demonstrating Myotally’s accuracy in quantifying protein expression (Figure 6F).

These validations confirm that Myotally provides accurate and reliable measurements of muscle fiber characteristics across various experimental settings.

## Limitations

The accuracy of the quantification heavily depends on image quality, with low-quality images requiring more extensive processing. Correct specification of the pixel to μm^2^ ratio is crucial for accurate cross-sectional area (CSA) measurements, and the default size parameters are optimized for 10X magnification with a 1:1 pixel to μm^2^ ratio from 7 DPI TA muscles in mice, as this measurement is commonly used in muscle injury research. Size range adjustments are necessary for other conditions. Manual verification may be needed for low-quality images, and managing multiple output files per image can be cumbersome. The protocol is specific to muscle tissue and requires user expertise in image processing and histological analysis for successful implementation and accurate results. Validation of myofiber MFI quantification was carried out using ectopic RFP expression, and users should interpret data carefully when using Myotally to quantify endogenous myofiber protein expression.

## Troubleshooting

### Problem 1

Step 12. Myotally outputs fiber sizes that are too small or too large.

### Potential solution

Optimal parameters can vary based on image magnification and extent of muscle repair. Adjust ‘Fiber size range (μm^2^)’.

### Problem 2

Step 12. Blank output files and empty folders.

### Potential solution

Output .txt files populate only after an image finishes processing, and some folders remain empty if intermediate images are still being processed. Verify processing status by checking if intermediate images are still being processed and check command prompt for the progress of image analysis. The intermediate images will be deleted as each folder is completely processed, leaving only a ‘final.tif’ image in each folder.

### Problem 3

Step 12. Long processing times.

### Potential solution

Processing times could be long with large images or when performing MFI calculations. Select high image quality option if the initial image quality is good. Additionally, users could crop large images into smaller tiles and run the software on these tiles to reduce processing time.

## Resource availability

### Lead contact

Dr. Thomas. A. Rando (trando@mednet.ucla.edu).

### Technical contact

Dr. Pieter Both (andriesboth@gmail.com).

Dr. Soochi Kim (soochikim@korea.ac.kr).

### Materials availability

Example images used in this study have been deposited to

https://github.com/pieterboth/Myotally/releases/tag/Myotally_V1.0.1.

https://zenodo.org/records/14197515.

### Data and code availability

We provide complete source code in our GitHub repository: https://github.com/pieterboth/Myotally/releases/tag/Myotally_V1.0.1.

## Acknowledgments

This work was supported by a training grant from the NIH (1 T32 GM 119995-1 A1) to the Stanford Stem Cell Biology and Regenerative Medicine PhD Program, a training grant from the Buck Institute for Research on Aging (T32AG000266-21) to D.I.B., a Korea University grant and the National Research Foundation of Korea (NRF) grant funded by the Korea government (no. RS-2021-NR060107) to S.K., and grants from the Department of Veterans Affairs (BLR&D and RR&D Merit Reviews) and the NIH (P01 AG036695, R37 AG023806, and R01 AR073248) to T.A.R.

## Author contributions

P.B. wrote the Myotally software program, designed the validation studies, and carried out experiments with assistance from C.W.N. T.A.R. provided guidance throughout. Manual measurements used for validation were done by S.K., J.K., M.A., D.I.B., C.W.N., and A.G. The results were interpreted by P.B. with guidance and input from T.A.R. The data were assembled and the paper was written by P.B., S.K., and T.A.R.

## Declaration of interests

The authors declare no competing interests.
