# Supplementary material for: Protocol for quantifying muscle fiber size, number, and central nucleation of mouse skeletal muscle cross-sections using Myotally software
Source: STAR Protoc. 2025 Jan 11;6(1):103555. doi: 10.1016/j.xpro.2024.103555 (PMC11772141; doi:10.1016/j.xpro.2024.103555)
Supplement: Document S1. Figure S1 [file mmc1.pdf]

Figure S1. Interpersonal variance for manual counting, related to Quantification and statistical analysis

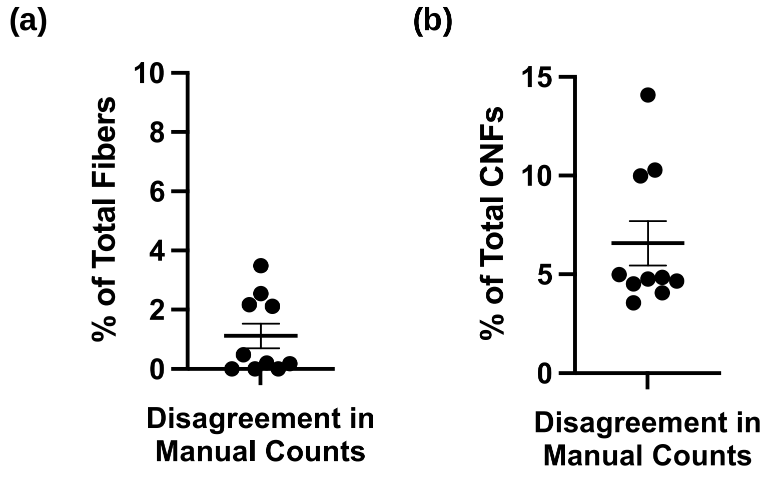

a) Percent of disagreement events in uninjured myofiber counts by Person 1 and Person 2. Any myofiber that was not counted by both counters constitutes a disagreement event. Percent disagreement was calculated for each image as $\frac{total\ disagreement\ events}{average\ total\ fibers} \times 100$ (n=10 image fields); error bars represent SEM.

b) Percent of disagreement events in CNF counts in injured muscle by Person.1 and Person 2. Any CNF that was not counted by both counters constitutes a disagreement event. Percent disagreement was calculated for each image as $\frac{total\ disagreement\ events}{average\ total\ CNFs} \times 100$ (n=10 image field); error bars represent SEM.
